# Improved Dermal and Transdermal Delivery of Curcumin with SmartFilms and Nanocrystals

**DOI:** 10.3390/molecules26061633

**Published:** 2021-03-15

**Authors:** Ralph W. Eckert, Sabrina Wiemann, Cornelia M. Keck

**Affiliations:** Department of Pharmaceutics and Biopharmaceutics, Philipps-Universität Marburg, Robert-Koch-Str. 4, 35037 Marburg, Germany; ralph-walter.eckert@pharmazie.uni-marburg.de (R.W.E.); sabrina.wiemann@pharmazie.uni-marburg.de (S.W.)

**Keywords:** curcumin, smartFilms, amorphous, nanocrystals, bead milling, dermal penetration, passive diffusion, stratum corneum, skin barrier

## Abstract

Poor aqueous solubility of active compounds is a major issue in today’s drug delivery. In this study the smartFilm-technology was exploited to improve the dermal penetration efficacy of a poorly soluble active compound (curcumin). Results were compared to the dermal penetration efficacy of curcumin from curcumin bulk suspensions and nanocrystals, respectively. The smartFilms enabled an effective dermal and transdermal penetration of curcumin, whereas curcumin bulk- and nanosuspensions were less efficient when the curcumin content was similar to the curcumin content in the smartFilms. Interestingly, it was found that increasing numbers of curcumin particles within the suspensions increased the passive dermal penetration of curcumin. The effect is caused by an aqueous meniscus that is created between particle and skin if the dispersion medium evaporates. The connecting liquid meniscus causes a local swelling of the stratum corneum and maintains a high local concentration gradient between drug particles and skin. Thus, leading to a high local passive dermal penetration of curcumin. The findings suggest a new dermal penetration mechanism for active compounds from nano-particulate drug delivery systems, which can be the base for the development of topical drug products with improved penetration efficacy in the future.

## 1. Introduction

**Figure 1:**
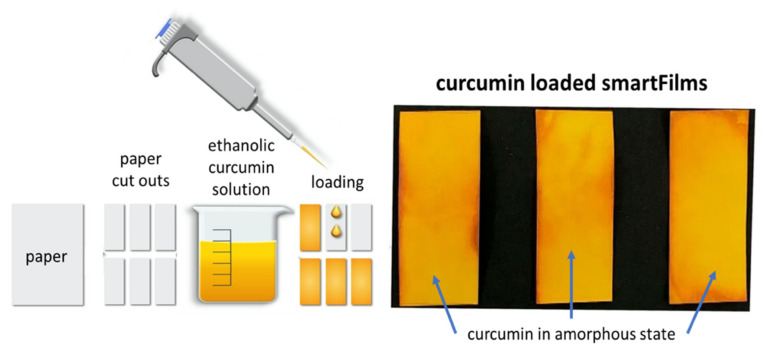
Scheme of smartFilm production.

Overcoming poor aqueous solubility and associated poor bioavailability is a major concern in drug delivery of small molecules [1]. Poor bioavailability is not limited to oral bioavailability but is also a challenge for effective topical drug delivery [2,3,4]. Various formulation strategies have already been developed to overcome this issue and the most successful formulation strategies so far include, e.g., micelles, liposomes, microemulsions, nanoemulsions, lipid nanoparticles, dendrimers, self-emulsifying drug delivery systems, solid dispersions, mixed crystals or the production of drug nanocrystals [5,6,7]. A recently developed formulation strategy for overcoming poor aqueous solubility is the smartFilm^®^ technology, where active ingredients (AI) are embedded in cellulose matrices in amorphous state [8,9]. The production of smartFilms is a simple three-step approach (Figure 1). First, the poorly soluble AI is dissolved in a solvent. Second, the obtained solution is applied on a cellulose matrix, e.g., commercially available paper, and finally, the solvent is evaporated. The so-obtained smartFilms retain the AI in the pores of the cellulose matrix and prevent the recrystallization of the AI. Consequently, due to the amorphous state of the AI, the kinetic solubility of the AI can be improved [10,11,12].

The advantage of the smartFilm technology for improved oral delivery was already shown in previous studies and recent studies could further demonstrate that the smartFilms can be transferred into tablets made from paper. Those tablets made from paper fulfil all criteria for conventionally produced tablets according to the European Pharmacopeia, e.g., hardness, friability, disintegration time. They also have a shiny and smooth surface and allow coating to make them easy to swallow. In fact, oral application of smartFilms is a highly promising approach for improved drug delivery of poorly soluble actives [10,11,12].

In addition to oral drug delivery, smartFilms can also be considered to be suitable for improved dermal drug delivery. After dermal application, the higher solubility of the AI is expected to build up a high concentration gradient of dissolved AI between smartFilm and skin. Thus, based on Fick’s law and, in comparison to classical formulations, an improved passive diffusion of the AI into or through the skin is assumed.

To date, a study that investigates the dermal penetration of AI from smartFilms is not available. Therefore, it was the aim of this study to investigate if smartFilms can improve the passive diffusion of poorly water-soluble AI into and through the skin after dermal application.

Curcumin, which is a well-known BCS class IV drug [13,14,15], was selected as model drug. Curcumin is well recognized for its multiple pharmacological activities, that include antioxidant, anti-inflammatory, antibacterial and antiviral activities [16,17]. Furthermore, it is a promising molecule for photodynamic therapy and dermal application of curcumin is described as promoting wound healing with no or only little scar formation [18,19]. The therapeutic use of curcumin is however limited and the major reason for this is its poor aqueous solubility, which hampers not only oral but also its topical bioavailability [13,14,15,16]. Therefore, to exploit the pharmacological potential of curcumin, drug delivery systems that increase not only oral but also its dermal penetration efficacy are of high interest. In this light, smartFilms loaded with curcumin seem to be a very promising formulation approach for an improved dermal penetration efficacy of curcumin.

In this study, curcumin-loaded smartFilms were produced, characterized regarding their physicochemical properties; their dermal penetration efficacy was determined in vitro and in the ex vivo porcine ear model. In order to evaluate the penetration efficacy of curcumin from the smartFilms realistically, curcumin bulk suspensions and curcumin nanocrystals with an approx. mean particle size of 200 nm were also produced and used as benchmark controls.

## 2. Results and Discussion

### 2.1. Production and Characterization of Curcumin-Loaded SmartFilms and Benchmark Controls

#### 2.1.1. Curcumin-Loaded SmartFilms

**Figure 2:**
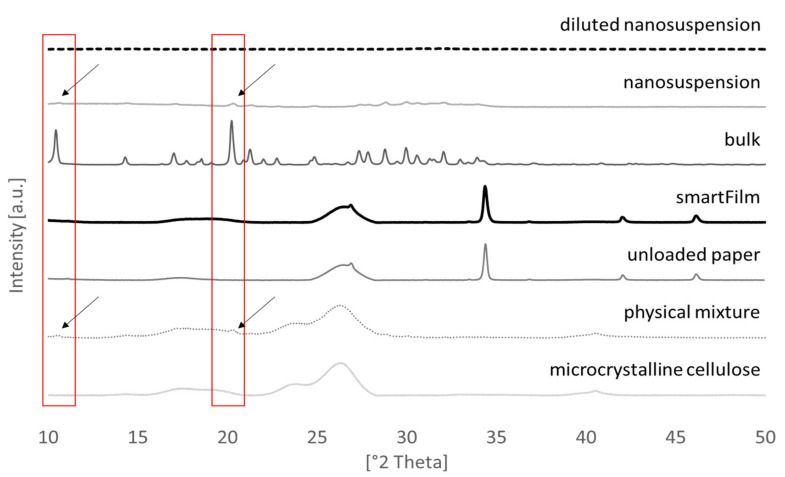
Results from XRD analysis.

Curcumin-loaded smartFilms were obtained upon the loading of paper cut outs with ethanolic curcumin solution (Figure 1—right). The crystalline state of the curcumin was determined by X-ray diffractometry from different spots of the smartFilms (Figure 2). Results revealed no crystalline reflexes for the smartFilms, indicating that curcumin was loaded in the smartFilms in amorphous state. The curcumin content was 684 µg per smartFilm (Table 1). This corresponds to an apparent curcumin load of 152 µg curcumin per cm^2^ matrix and thus is equal to the theoretical curcumin load of 150 µg curcumin per cm^2^ matrix.

#### 2.1.2. Curcumin Bulk- and Nanosuspensions

The curcumin bulk suspension possessed a particle size of about 45 µm, which was decreased to about 200 nm by nanomilling (Table 1). XRD analysis revealed that bead milling did not alter the crystalline state of curcumin and confirmed that the curcumin in the nanosuspension was still in crystalline state (Figure 2). The content of the curcumin was 466 µg/50 µL for the bulk suspension and 432 µg/50 µL for the nanosuspension (Table 1). This corresponded to apparent curcumin contents of 4.2% and 3.9% and to relative nominal curcumin contents of 105% and 97.5%, respectively. The slight decrease of curcumin content in the nanosuspension is a side effect of the production process due to adsorption of curcumin onto the surface of the milling beads. This loss accounted for about 7%.

Benchmark controls for the skin penetration studies with a similar amount of curcumin in 50 µL (=applied dose on skin) as the smartFilms were obtained by diluting the original curcumin suspensions. After dilution, the particle size of the bulk suspension was significantly decreased, whereas only very minor changes in size were detected for the nanosuspension (Table 1). X-ray analysis showed no reflexes for the diluted nanosuspension (Figure 2), indicating that high-pressure homogenization (HPH) transferred the curcumin nanocrystals into an amorphous state after dilution [20].

#### 2.1.3. Dissolution Behavior

**Figure 3:**
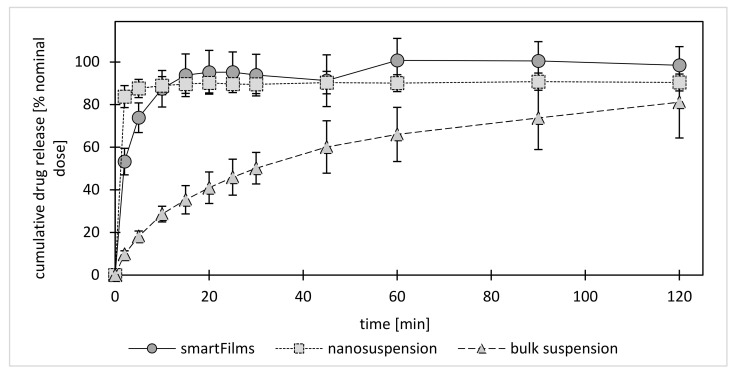
Dissolution profiles of curcumin from smartFilms and benchmark controls (curcumin bulk suspension and nanosuspension—explanations—c.f. text).

The dissolution (release) of curcumin from the bulk material was relatively slow. Only 33% of the curcumin was released after 15 min. After 1 h about 66% and after 2 h about 80% of the curcumin was released (Figure 3). The release of curcumin from the nanocrystals was fast, i.e., 90% of the curcumin was already released after 5 min. The results demonstrate the well-known effect of nanosized drug crystals on the kinetic solubility and the dissolution rate of poorly water-soluble compounds [21,22,23,24,25]. Despite this, data of the present study also provide evidence that already small quantities of larger sized particles within a nanosized formulation can affect the overall release of the active compound. Hence, the 10% of non-dissolved curcumin in the nanosuspension can be attributed to the 10% of larger sized particles within this formulation (Table 1). The d(v)0.9 indicates that 90% of the volume of the particles is smaller or equal to the given value [26]. The d(v)0.9 of the nanosuspension was 230 nm, i.e., 90% of the volume of the particles was equal or below this size value (Table 1). These small particles were able to dissolve, whereas the larger sized particles (up to 2.2 µm) with resulting lower dissolution rate and lower kinetic solubility remained undissolved.

In comparison to the nanosuspension, the curcumin release from the smartFilms was slightly retarded (Figure 3). The slower onset of the release can be attributed to the structure of the paper, because the first minutes of the release experiments are required to wet the dry paper matrix. Only after sufficient wetting of the paper does the curcumin come into contact with its solvent. After contact with the solvent the amorphous curcumin is instantly dissolved and thus exhaustively released from the paper matrix (Figure 3). Hence, the amorphous state of the curcumin in the smartFilms enabled a complete release of 100% of curcumin within only 1 h (Figure 3).

### 2.2. Determination of Dermal Penetration Efficacy—Ex Vivo

**Figure 4:**
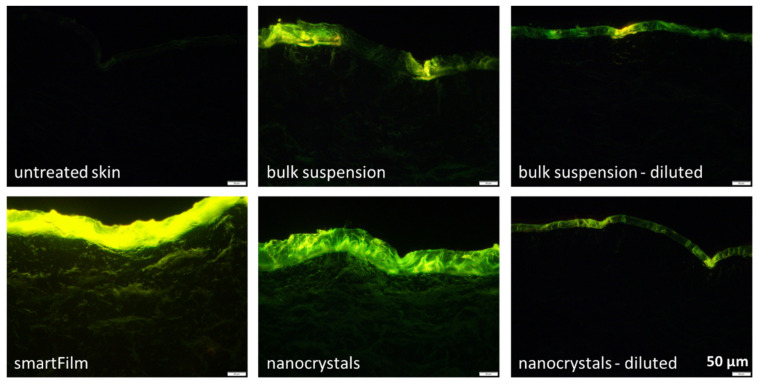
Comparison of dermal penetration efficacy of curcumin from bulk material, nanocrystals, smartFilms and from diluted bulk material and diluted nanocrystals to obtain similar amounts of curcumin on the skin as for the smartFilms. Images (200-fold magnification) were obtained by epifluorescence microscopy from punch biopsies of porcine ears (penetration time for the formulations was 6 h at 32 °C: for more details—c.f. Supplementary Materials S1).

In the next step, the dermal penetration efficacy was determined on the ex vivo porcine ear model [27,28,29]. The smartFilms resulted in an efficient permeation of curcumin into the stratum corneum (SC) and a pronounced transdermal penetration into deeper layers of the viable cells of the dermis (Figure 4). This data confirmed the theory and provided first scientific evidence that the smartFilms are indeed a highly effective formulation principle for efficient transdermal delivery of poorly soluble compounds like curcumin.

In contrast, only a weak uptake of curcumin into the SC and no transdermal penetration was observed for the curcumin bulk- and nanosuspensions that contained similar curcumin contents as the smartFilms (Figure 4, diluted formulations). The observed poor curcumin penetration was expected for the bulk material, since mean particle sizes of about 25 µm were present, resulting in low solubility (c.f. Section 3.1). However, the poor penetration of curcumin from the nanosuspension with excellent solubility was not expected. The higher concentration of dissolved curcumin in the nanosuspension should have created a higher concentration gradient between formulation and skin and thus, should have led to a higher passive penetration of the active compound from the formulation into the skin [30].

**Figure 5:**
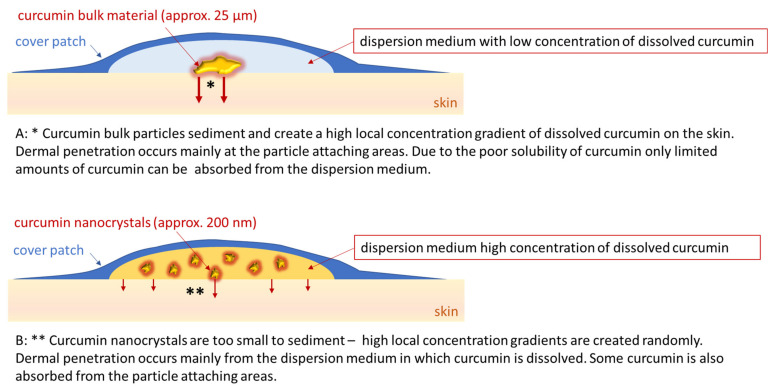
Principle of dermal penetration of curcumin from curcumin bulk suspensions (**A**) and curcumin nanocrystals (**B**).

A possible explanation for this unexpected observation might be sedimentation of the bulk material in the suspension. Upon sedimentation, the larger particles in the bulk material come into direct contact with the skin. This would create a high local concentration gradient of curcumin that dissolves from the surface of the particles (Figure 5, upper). In contrast, no sedimentation would occur with particles in the nanometer range [31]. Thus, despite the higher concentration gradient between formulation and skin in the whole formulation, the nanocrystals would not be in close contact with the skin and no high local concentration gradients would be built up. Consequently, this would result in a less efficient uptake of curcumin into the skin (Figure 5, lower). The theory of sedimented larger particles in the bulk suspension is supported by the epifluorescence microscopic images (Figure 4). The application of the bulk suspension resulted in some bright spots on the skin, indicating that the skin areas were in contact with large curcumin crystals. In contrast, the skin treated with the nanocrystals appeared more homogeneous on epifluorescence microscopic images (Figure 4).

### 2.3. Determination of Dermal Penetration Efficacy—Digital Image Analysis

**Figure 6:**
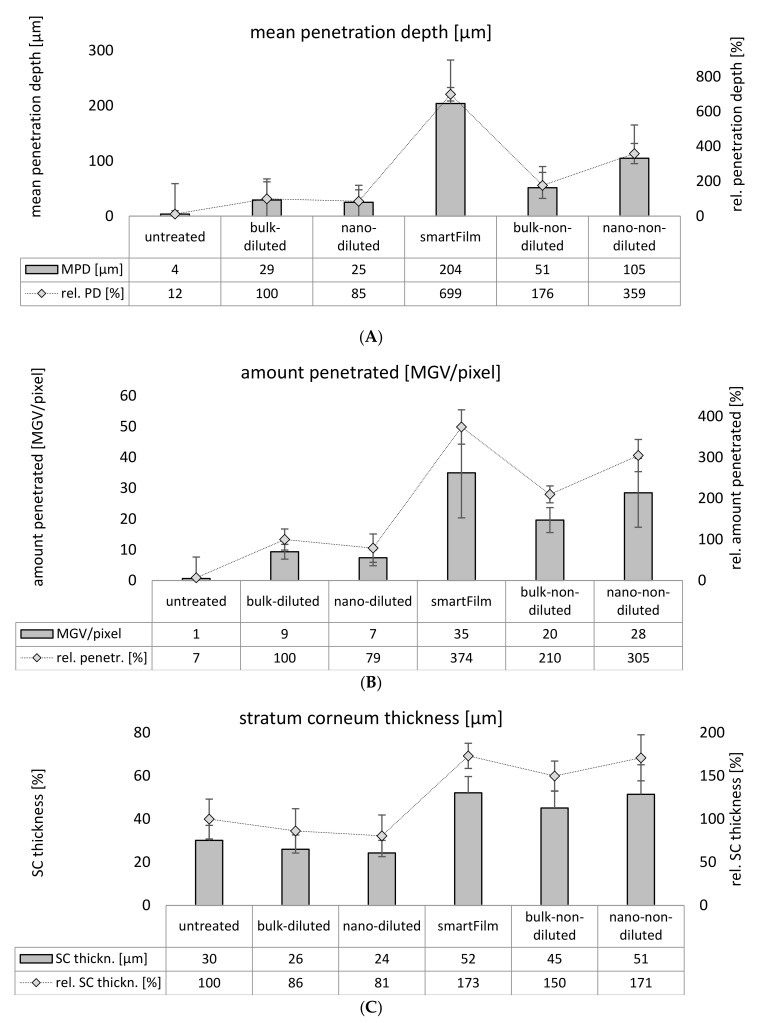
Dermal penetration efficacy of curcumin from smartFilms and benchmark controls: (**A**)—mean penetration depth, (**B**)—total amount of penetrated curcumin, expressed as mean gray value (MGV/pixel), (**C**)—thickness of stratum corneum.

Digital image analysis substantiated the results obtained from visual image inspection (Figure 6). The penetration depth of curcumin from the smartFilms was about 200 µm and was <30 µm for the diluted bulk- and nanosuspensions with similar curcumin contents (Figure 6A). The mean gray value (MGV), which is used as a surrogate for the total amount of penetrated curcumin, was 35 for the smartFilms, but only 9 and 7 for the diluted bulk- and the nanosuspension (Figure 6B). Hence, the penetration efficacy of curcumin was about 7-fold higher than the smartFilms when compared to the diluted curcumin bulk- and nanosuspensions.

Despite this, the data also show a lower penetration depth and a lower amount of penetrated curcumin from the nanocrystals when compared to the bulk suspension. The differences were not significant, but the observed trend further supports the theory of sedimented larger particles from the bulk suspensions and less local contact points for the nanocrystals.

### 2.4. Determination of Dermal Penetration Efficacy—Franz Diffusion Cells

**Figure 7:**
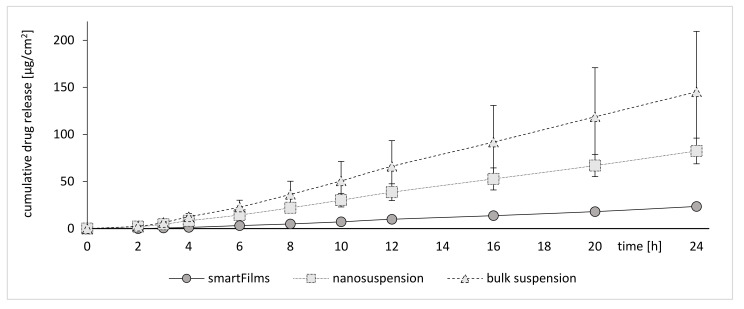
In vitro Franz cell diffusion tests to determine the passive diffusion of curcumin from smartFilms and the benchmark controls (curcumin bulk and nanosuspension).

The results obtained from Franz diffusion cell experiments further supported the theory of sedimented larger particles from the bulk suspensions. In addition, in this setup the bulk material led to the most pronounced penetration of curcumin, whereas the nanosuspensions were less effective (Figure 7). Interestingly, it was observed that the smartFilms, which were demonstrated to be the most effective formulation in the ex vivo model, were the least effective in the in vitro setup (c.f. Figure 4, Figure 6 and Figure 7).

The poor biopredictivity and the misleading results obtained from the Franz diffusion cell experiments can be explained with the experimental setup, which requires the use of an acceptor media which provides sink conditions for the active ingredient. During the experiment the acceptor medium is in direct contact with the diffusion membrane and ensures constant moistening of the membrane [32].

If particles of the active compound come into contact with the diffusion membrane, they will also come into contact with the acceptor medium. In this case, the acceptor medium accelerates the dissolution of the particles and creates a high concentration gradient, leading to an accelerated diffusion of curcumin through the membrane. The effect is most pronounced for the bulk suspension with large settling particles and is less pronounced for the nanocrystals, which will not sediment due to their small size. For the smartFilms the effect is least pronounced, since curcumin is embedded into the matrix of the paper and thus, cannot come into direct contact with the diffusion membrane. The sedimentation process of the large particles from the bulk suspension occurs randomly, which becomes obvious in the high standard deviations (Figure 7). In contrast, the penetration of curcumin is more homogeneous for the nanocrystals and the standard deviation is much lower when compared to the bulk suspension. The diffusion of the curcumin from the smartFilms is highly reproducible and results in the smallest standard deviations (Figure 7).

The release of curcumin from the smartFilms seemed to follow a zero-order release kinetic. However, when transferring the data into the Korsmeyer–Peppas model, it was found that the release exponent was >1, indicating that the release of curcumin from smartFilms is not a simple case II (zero-order kinetic release) but rather a super case II transport [33,34]. This means that the release rate of curcumin is slightly increasing with time. The reason for this is related to the swelling of the paper matrix and a subsequent relaxation of the paper matrix after its complete wetting. The results are in line with the data obtained from the release experiments (c.f. Figure 3), because also in this experiment a slightly retarded release of the curcumin was found for the smartFilms in comparison to the curcumin nanocrystals.

### 2.5. A Step Forward to Understanding Passive Dermal Penetration of AIs from Particle Containing Topical Formulations

Data of this study show that passive dermal penetration of an AI is not only driven by the amount of dissolved AI in the dispersion medium, i.e., in the vehicle, when the formulation contains particles that can come into close contact with the skin. The here observed penetration-enhancing effect of particles could be exploited to improve the passive dermal penetration of active compounds by simply adding particles to topical formulations in the future. However, if the contact of particles to the skin only occurs in an in vitro or ex vivo test assay (for example, due to sedimentation of large particles in an infinite dose setup), but not in a real in vivo situation, in vitro and ex vivo dermal penetration testing of liquid suspensions can be misleading. Therefore, based on the data of the present study, it is suggested to test dermal formulations that contain particles only in setups that mimic real in vivo situations.

Based on the results, it was also hypothesized that an increase in the number of curcumin particles within a formulation should also increase the number of particles being in direct contact with the skin. The higher number of particles being connected to skin should then lead to an increased penetration of curcumin. The theory was tested by applying the original non-diluted curcumin suspensions on fresh porcine ears (Figure 4 and Figure 6). Based on textbook knowledge, only the concentration of dissolved active contributes to the dermal passive diffusion of an AI. Hence, no changes in the dermal penetration should occur if only the number of undissolved particles is increased in a topical formulation while the number of dissolved molecules is kept unchanged.

**Figure 8:**
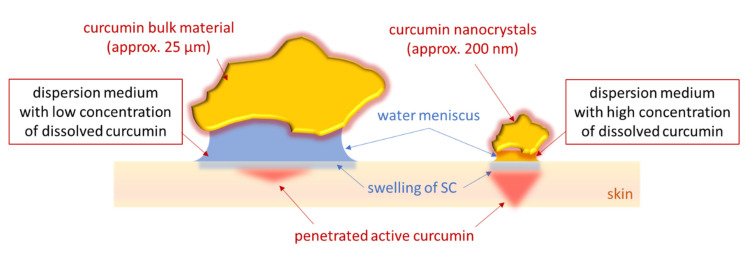
Scheme of suggested mechanism of passive dermal penetration of active compounds from particle-containing formulations (explanations c.f. text).

The original non-diluted curcumin suspensions contained an about 4-fold higher particle concentration than the diluted suspensions (c.f. Table 1). The increase in particle concentration and the larger particle size of the bulk suspension increased the dermal penetration of curcumin tremendously (Figure 4 and Figure 6). When compared to the diluted suspensions, the penetration depth and the total amount of penetrated curcumin were approximately doubled for the non-diluted curcumin bulk material and were about 4-fold higher for the non-diluted curcumin nanosuspensions. These results provide first evidence that the number of drug particles in a vehicle influences the dermal penetration efficacy of AI into the skin. Hence, data prove that increasing amounts of drug particles within a topical formulation can tremendously increase the penetration depth and the total amount of penetrated AI into and through the skin. Interestingly, it was also found that the increase in particle concentration increased the thickness of the stratum corneum (Figure 6C). After 6 h penetration time, the untreated stratum corneum possessed a thickness of about 30 µm. The thickness of the SC was significantly decreased when the skin was treated with the diluted suspensions, indicating that these suspensions caused no SC swelling or occlusive effect on the skin. Data also show that the patch that was applied over the smartFilms to fixate them and that was also used to cover the curcumin suspensions to provide a comparable application situation also had no occlusive effect on the SC. Hence, the patch was waterproof from outside but highly permeable for liquids located between skin and patch, as described by the manufacturer. The non-diluted bulk suspension increased the SC thickness by about 50%. The smartFilms and the non-diluted nanocrystals increased the SC thickness by about 170% when compared to the untreated skin (Figure 6C). The occlusive effect was reasonable for the smartFilms because the cellulose matrix shows properties of a sponge by retaining water which can continuously hydrate the skin, thus leading to the observed swelling of the SC. However, an occlusive effect caused by drug crystals as seen for the bulk- and nanoparticle suspensions was not expected and was—to our best knowledge—not yet described in the literature. Nonetheless, it seems reasonable that the adhesion of particles to the skin from liquid vehicles occurs by a creation of an aqueous meniscus between particle and skin (Figure 8). This aqueous meniscus would then cause a local swelling of the SC. The effect seems to be non-measurable if only a small number of particles is attached to the skin but seems to increase with an increasing number of particles being connected to the skin. Similar to an occlusion effect, this local hydration of the SC can be considered to foster the local dermal penetration of the active compounds.

The here observed particle-assisted improved passive dermal penetration can be explained by two effects. The first effect is caused by the capillary forces [35] of the aqueous meniscus that fixate the particles to the skin, thus increasing their retention time on the skin (Figure 8). The second effect is the liquid in the meniscus which provides a reservoir for dissolved drug molecules from which a constant penetration of curcumin into the skin can occur (Figure 8). Penetrated drug molecules that left the liquid reservoir of the meniscus can be replaced by molecules that are released from the curcumin particle. By this, a high local concentration gradient of dissolved curcumin between the aqueous meniscus and the SC can be created and maintained. Similar to the classical penetration principle of drug compounds from semi-solid formulations, which follows Fick’s law, this local drug penetration can also be considered to be dependent on the main parameters of Fick’s law, i.e., contact area, concentration gradient and diffusion coefficient.

The contact area depends on particle size and the total number of particles. The concentration gradient depends on the amount of dissolved active in the vehicle and can be probably altered by the type of dispersion medium and/or by the particle size of the drug particles. The local diffusion coefficient can be considered to be influenced by the swelling of the SC, meaning that a more intense swelling of the SC and/or a large area of a locally swollen SC will increase the local diffusion coefficient. Consequently, the local diffusion coefficient can be increased by a large number of particles that can create a liquid meniscus between particle and skin. In addition, it can be assumed that the type of vehicle in which the particles are suspended will also have an impact on the local diffusion coefficient. The meniscus between particle and skin can only be formed if the vehicle allows the drug particles to come into contact and to remain in contact with the skin. In addition, the maintenance of the meniscus between particle and skin will probably also be dependent on the film-forming properties of the vehicle, i.e., the ability of the vehicle to maintain sufficient moisture, which prevents the drying out of the formulation and the meniscus. Hence, vehicles that contain humectants can be considered to enable a longer retention of an aqueous meniscus than formulations that contain excipients which are prone to a fast evaporation after topical application.

Based on these considerations, formulations that contain high amounts of small-sized particles with high kinetic solubility and dissolution rate that are suspended in thixotropic vehicles containing humectants should especially enable highly efficient dermal and transdermal penetration of poorly soluble actives. The thixotropic behavior would cause the vehicle to become fluid during the application on the skin and allow the particles to come into contact with the skin and to form the aqueous meniscus between particle and skin. The thixotropic vehicle would solidify after the end of application and would enable the particles to remain in contact with the skin. The moisturizer would prevent drying out and would enable the fixation of the particles. Thus, the local swelling of the SC would be enabled, as well as the creation and maintenance of the high local concentration gradient (Figure 8).

**Figure 9:**
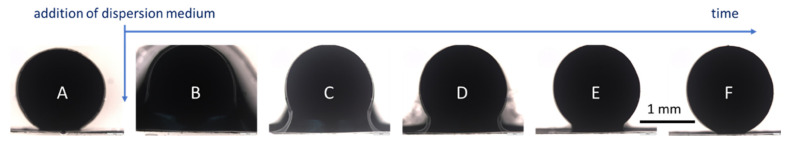
Microscopic images (magnification 100-fold) of a zircon dioxide bead connected to a glass surface via an aqueous meniscus that was formed upon the addition of surfactant solution. Detailed explanations c.f. text.

The creation of an aqueous meniscus that forms between particles and a surface is well-described in literature [35,36] and was also demonstrated here by placing zircon oxide milling beads (c.f. 2.2.2.) next to a microscopic slide that was placed in 90° direction on another microscopic slide. The arrangement was inspected by light microscopy and subsequently a small amount of surfactant solution (1% Plantacare solution, c.f. 2.2.2) was added (Figure 9). Initially, the surfactant solution formed a large droplet around the beads, mimicking a vehicle with suspended particles (Figure 9B). Over time, the surfactant solution evaporated and was also soaked in between the interspace of the two microscopic slides. The loss in surfactant solution resulted in the formation of the proposed aqueous meniscus between particle and glass surface (Figure 9C). The meniscus decreased with decreasing surfactant volume (Figure 9D,E) and finally disappeared (Figure 9F). With the disappearance of the meniscus between the glass surface and the bead, the capillary forces between bead and glass surface were also lost. Thus, the bead was not connected to the glass surface anymore and was easy to move away from it.

The results substantiate the theory that particles can connect to the skin via the formation of an aqueous meniscus and show that the meniscus is important for maintaining the connection between the particle and the skin. The observation can be further supported by a very recent study by Pelikh et al. that investigated the influence of the vehicle type on the penetration efficacy of curcumin from nanocrystals and the ability to transport nanocrystals into hair follicles. In this study, it was shown that the addition of humectants, e.g., glycerol, increased the passive dermal penetration of curcumin from curcumin nanocrystals, whereas the addition of ethanol decreased it. The results were not conclusive at that time because ethanol is known to act as a penetration enhancer. However, the results can now be explained by the meniscus theory. The ethanol evaporates more quickly than pure water and thus the connection between skin and particle is disrupted earlier when compared to the aqueous nanosuspensions without added ethanol. Consequently, a less efficient penetration is obtained for the ethanol-containing vehicles in comparison to vehicles that consist of water without ethanol. The addition of glycerol, which is hygroscopic, can further delay the evaporation of water. This leads to a longer maintenance of the aqueous meniscus between particle and skin and to a more pronounced local swelling of the SC. Thus, this promotes the passive dermal penetration of curcumin when compared to pure water [37].

The here proposed mechanism of dermal drug absorption from topical formulations with suspended drug particles now needs further proofs from more detailed and mechanistic studies. Nevertheless, data so far are already sufficient to conclude that dermal drug delivery with suspended drug particles is not only dependent on the amount of dissolved AI in the vehicle. Results rather show that the number of particles, the size of the particles and the ingredients of the vehicle can also have a tremendous effect on the penetration efficacy of dermally applied AI. All these parameters need to be addressed during formulation development of topical drug products and can be adjusted accordingly to obtain formulations with improved dermal penetration efficacy.

## 3. Materials and Methods

### 3.1. Materials

Curcumin is a BSC class IV drug and possesses a strong autofluorescence [38,39,40]. Due to these properties, it was used as model drug in this study. Curcumin was used as *curcuma longa* extract powder (purity = 95% curcuminoids, curcumin content 80%, Receptura Apotheke—Cornelius-Apothekenbetriebs-OHG, Frankfurt am Main, Germany). Decyl glucoside (Plantacare^®^ 2000UP, PLC 2000) was used as stabilizer for the curcumin suspensions and was kindly provided by BASF, Ludwigshafen am Rhein, Germany. Purified water was used as dispersion medium and was obtained from a PURELAB^®^ Flex 2 water purification system (ELGA Labwater, Veolia Water Technologies Deutschland GmbH, Celle, Germany).

### 3.2. Methods

#### 3.2.1. Production of SmartFilms

The smartFilms were manufactured according to a previously developed protocol [10,12]. Printer paper (juwel80 premium, Stora Enso, Stockholm, Sweden) was used as matrix because of its smooth surface without embossments to ensure a close contact to the skin and Ethanol 96% (*v*/*v*) (HPLC grade, VWR International GmbH, Darmstadt, Germany) was used as solvent for curcumin. Ethanolic curcumin solutions containing 2 mg/mL pure curcumin were prepared by dissolving 125 mg curcumin extract in ethanol up to 50.0 mL. The paper was cut into rectangles with a size of 3 cm × 1.5 cm (4.5 cm^2^) and was loaded with curcumin by dropping 50 µL of the ethanolic curcumin solution onto the surface of the paper with a pipette. The matrix was dried at room temperature and the procedure continued until a nominal content of 150 µg curcumin/cm^2^ paper was achieved (Figure 1).

#### 3.2.2. Production of Curcumin Bulk- and Nanosuspensions

A curcumin bulk suspension and a curcumin nanosuspension were used as benchmark controls. The curcumin suspensions contained 5% (*w*/*w*) curcumin extract and 1% (*w*/*w*) surfactant. The bulk suspension was prepared by adding the curcumin bulk material to the surfactant solution while stirring. The nanosuspension was obtained by subjecting the bulk suspension to small-scale bead milling [41]. For this, the bulk suspension was filled into a 25 mL Erlenmeyer flask to which a magnetic stirring bar (ASTEROID 25, 2mag AG, München, Germany) and yttrium stabilized zirconium oxide beads (SiLibeads^®^, Sigmund Lindner GmbH, Warmensteinach, Germany) with a diameter of 1–1.2 mm were added to yield a bead: suspension ratio of 40: 60 (*v*/*v*). The mixture was bead milled for 6 h at 1000 rpm on a magnetic stirrer (IKA^®^ RET basic, IKA^®^, Staufen im Breisgau, Germany). The temperature was constantly kept below 5 °C.

The obtained original suspensions were analyzed regarding their accurate curcumin content and then diluted with surfactant solution (1% (*w*/*w*) PLC 2000) until comparable curcumin contents to the smartFilms were received. The accurate dilution factor being necessary depended on the accurate amount of curcumin in each formulation and was therefore calculated individually. The freshly diluted nanosuspensions were immediately subjected to high-pressure homogenization (Avestin EmulsiFlex-C5, Avestin Inc., Ottawa, ON, Canada, 10 cycles at 1000 bar) to avoid Ostwald ripening due to a partial dissolution of smaller sized nanocrystals [42]. All formulations produced were characterized at the day of production and prior to dermal application. In between they were stored in the refrigerator at 5 °C ± 2 °C.

#### 3.2.3. Characterization

All formulations were characterized regarding crystalline state of the curcumin, curcumin content and dissolution profile. In addition, particle size analysis was performed for the curcumin bulk material and the curcumin nanocrystals.

##### Determination of Crystalline State

The crystalline state was analyzed by powder X-ray diffraction (XRD) using an X’Pert Pro MPD diffraction system (Malvern Panalytical Ltd., Malvern, UK) with a diffraction angle 2θ range of 10° to 50° and a scan rate of 0.013°/s. The X-ray source was a cobalt anode, operated at a tension of 40 kV and a current of 35 mA. The XRD diffraction pattern of the unprocessed curcumin served as crystalline, positive control and a physical mixture of curcumin in microcrystalline cellulose (Comprecel^®^ Mingtai chemical Co., LTD, Taoyuan City, Taiwan) with the same content of curcumin as in the smartFilms served as control for the limit of detection for crystalline material in the cellulose-based matrices. Unloaded paper and microcrystalline cellulose served as negative controls. Prior to the measurements, the viscosity of the liquid formulations (curcumin bulk- and nanosuspensions) was increased with 1.5% (*w*/*w*) locust beam gum to form a gel that inhibits the movement of the crystals during the measurement, which would disturb the diffraction pattern [43]. 

##### Determination of Curcumin Content

The curcumin content was determined by high-performance liquid chromatography (HPLC). The analysis was performed with a validated HPLC method using an Agilent 1260 Infinity II HPLC system (Agilent Technologies Inc., Berlin, Germany), equipped with a quaternary pump VL (G7111A), autosampler (G7129A) and a diode array detector (G7117C DAD HS). Separation of the analyte was carried out with a C18-reversed phase fused-core column (Ascentis^®^ Express, 75 mm × 4.6 mm, 2.7 µm particle size, Merck, Darmstadt, Germany) equipped with a complementary guard column (Ascentis^®^ Express, Merck, Darmstadt, Germany) under isocratic elution (60:40 (*v*/*v*) 0.01 M phosphoric acid: acetonitrile, pH 2.2) and at a flow rate of 1 mL/min. Temperature was set to 40 °C and the detection wavelength was 425 nm. The injection volume was 2.5 µL and the run time was set to 10 min. The range of the method was 0.125 µg/mL (= limit of quantification, LOQ) to 80 µg/mL with a seven-point calibration line and a linear correlation coefficient of r^2^ = 0.9999. The limit of detection (LOD) was 0.05 µg/mL. Further details are displayed in the Supplementary Materials Section S2.

##### Determination of Dissolution Profile

The dissolution profiles of curcumin from the different formulations were assessed according to the test method 2.9.3. of the European Pharmacopeia 9.0 (Pharm. Eur.) using the paddle apparatus (PTWS 120D, Pharmatest, Hainburg, Germany). The dissolution medium was 500 mL of 5% (*w*/*w*) sodium dodecyl sulphate solution (SDS, Carl Roth GmbH, Karlsruhe, Germany) adjusted to a pH of 5.5 with phosphoric acid (Merck, Darmstadt, Germany). SDS was added to increase the maximum solubility of curcumin, thus generating sink conditions. A pH of 5.5 was adjusted to prevent basic degradation of curcumin [44]. The temperature was constantly kept at 37 °C ± 0.5 °C and the paddle speed was set to 100 rpm. Samples were taken after 2, 5, 10, 15, 20, 25, 30, 45, 60, 90 and 120 min and the withdrawn volume was replaced with fresh medium. Subsequently, all samples were filtered through a 0.22 µm syringe filter (Pall, Dreieich, Germany) and the filtrate was subjected to HPLC analysis. All formulations were tested in triplicate.

##### Size Characterization of Curcumin Suspensions

Size characterization was performed by three independent techniques. Dynamic light scattering (DLS, Zetasizer Nano ZS, Malvern Panalytical Ltd., Malvern, UK, data analysis with the general-purpose mode) was used to determine the hydrodynamic particle size diameter (z-average) and the polydispersity index (PdI) of the nanocrystals. Laser diffraction (LD, Mastersizer 3000, Malvern Panalytical Ltd., Malvern, UK) was used to detect possible larger sized particles. The median volumetric diameters of the suspensions were analyzed with Mie-theory. The real refractive index was set to 1.87 and the imaginary refractive index was separated for the blue light (470 nm) and the red light (632.8 nm) laser beam. The UV/Vis spectrum of curcumin shows absorption at a wavelength of 470 nm and no absorption at 632.8 nm. Thus, the imaginary refractive indices were set to 0.1 (blue light) and 0.01 (red light). Light microscopy (Olympus BX53, Olympus Corporation, Shinjuku, Japan), equipped with an Olympus SC50 CMOS color camera (Olympus soft images solution GmbH, Hamburg, Germany) was also performed to gain further, more detailed information on the shape, size and size distribution of the particles.

#### 3.2.4. Determination of Dermal Penetration Efficacy of Curcumin

The dermal penetration was assessed in vitro with Franz diffusion cells and ex vivo on a porcine ear model.

##### In Vitro Testing with Franz Diffusion Cells

The in vitro penetration kinetics of curcumin from the different formulations were determined with Franz diffusion cells with a nominal volume of 12 mL. The acceptor medium used was a 0.1 M citrate buffer pH 5.0 with ethanol (50/50 (*v*/*v*)). Ethanol was added to generate infinite dose conditions (c_t_ << c_s_) for testing [45]. After the setup of the diffusion cells, the smartFilms, which were manufactured on circular paper to fit exactly onto the test area of the Franz diffusion cells, were wetted with purified water and placed on a Strat-M^®^ membrane for transdermal diffusion testing (Merck, Darmstadt, Germany). The diffusion area was 2.2 cm^2^, the temperature was set to 32 °C ± 0.5 °C and the penetration time was 24 h. Samples were withdrawn at predefined time points and replaced with fresh medium. The curcumin content was determined by HPLC analysis and the experiment was performed in triplicate. Curcumin bulk- and nanosuspensions were tested in similar setup, while the amount of curcumin in the suspension added to the donor compartment was similar to the amount of curcumin loaded in the smartFilms. The accurate amount needed for the bulk- and the nanosuspensions was calculated based on the exact curcumin content of each formulation, individually assessed by HPLC analysis.

##### Ex Vivo Skin Penetration Studies

In addition, the dermal penetration of curcumin from the different formulations was investigated on the ex vivo porcine ear model [27]. Fresh ears were obtained from a local slaughterhouse, cleaned with lukewarm tap water, and carefully dried with paper tissue. Intact skin areas (3 cm × 1.5 cm) without visible scratches or wounds were selected, marked, and all hairs within these areas were cut short to a length of about 1–3 mm. Prior to application, the smartFilms were wetted with purified water, placed on the skin, and fixed to the skin with waterproof but breathable patches (DracoPor waterproof, Dr. Ausbüttel & Co. GmbH, Dortmund, Germany). For the curcumin suspensions, 50 µl was applied on the porcine skin and homogeneously distributed throughout the whole skin area with a saturated glove finger [46]. Afterwards, the formulations were also covered with waterproof patches to yield similar penetration conditions as for the smartFilms. The formulations could penetrate into the skin for 6 h, stored at 32 °C in an oven. Afterwards punch biopsies (Ø = 15 mm) were taken, immediately embedded in Tissue-Tek^®^ (Sakura Finetek Europe B.V., Alphen aan den Rijn, The Netherlands), and frozen at −80 °C until further use. Each formulation was tested on three different independent ears and in triplicate.

##### Epifluorescence Microscopy

Subsequently, the skin biopsies were sectioned into 20 µm thick vertical cuts (cryomicrotome Mod. 2700, Reichert-Jung, Nußloch, Germany) and the autofluorescence of the curcumin in the skin sections was visualized by inverted epifluorescence microscopy (Olympus CKX53, equipped with an Olympus DP22 color camera, Olympus Life Science Solutions GmbH, Hamburg, Germany). In order to dim the autofluorescence of the skin to a minimum, the intensity of the fluorescence light source (130 W U-HGLGPS illumination system, Olympus Deutschland GmbH, Hamburg, Germany) was set to 50% and the exposure time was 50 ms. The filter selected for analysis of the skin sections was the DAPI HC filter block system (excitation filter: 460–490 nm (BP), dichroic mirror 500 nm, emission filter: starting at 500 nm (LP)).

##### Digital Image Analysis

The fluorescence images (200-fold magnification) were subjected to digital image analysis with ImageJ software [47,48] to determine the mean penetration depth (MPD) of curcumin and the mean gray value (MGV) as a surrogate for the total amount of penetrated curcumin into and through the skin [27,37,49]. A possible swelling of the stratum corneum (SC) was also investigated by determining the mean SC thickness. The SC thickness and the penetration depth were directly assessed with the ImageJ scale function, after the scale of the images (50 µm) was set to 2.84 pixel/µm. The MGV was measured from each image and represents the mean light intensity/pixel within this image. In order to use the MGV as surrogate for the total amount of penetrated curcumin, prior to the MGV measurements, images were subjected to an auto threshold algorithm (Supplementary Materials Section S2) to subtract the remaining autofluorescence of the skin from all images. After the elimination of the skin autofluorescence (class II pixel), the remaining light intensity within the image corresponds to curcumin in the skin biopsy (class I pixel). Consequently, the measured MGV value can be used as a surrogate for the total amount of penetrated curcumin into the skin [27,37,49].

#### 3.2.5. Statistical Analysis

Statistical analysis was performed with JASP software, version 0.13.1 [50]. Normal distribution and variance homogeneity of the data were tested with the Shapiro–Wilk and the Levene’s test, respectively. For the normally distributed data, the mean values were compared by a one-way ANOVA that was Welch-adopted in case of variance heterogeneity. Tukey and Games-Howell post-hoc tests were performed to compare the mean values between each other. For the non-parametric data sets a Kruskal–Wallis analysis of variance with Dunn’s post-hoc tests were performed [51]. Bonferroni–Holm correction was used to account for alpha error accumulation. *p*-values < 0.05 were considered statistically significant.

## 4. Conclusions

Curcumin-loaded smartFilms were produced and characterized in this study. Results demonstrated the loading of the curcumin into the cellulose matrix in amorphous state which resulted in an increased dissolution rate and increased kinetic solubility when compared to curcumin bulk material. According to the Korsmeyer–Peppas model, the release of the curcumin from the paper matrix follows a biphasic super case II transport. The first phase is the wetting of the cellulose fibers with a slow release of curcumin. The second phase follows a zero-order release and occurs after the complete wetting of the cellulose fibers.

SmartFilms were demonstrated to enable a pronounced dermal and transdermal penetration of curcumin. Thus, it was confirmed that smartFilms are a very potent drug delivery system for efficient dermal and transdermal delivery of poorly water-soluble active compounds. The dermal and transdermal penetration of curcumin from smartFilms was compared to curcumin nanocrystals that were used as benchmark controls. The smartFilms were superior when compared to nanocrystals with similar curcumin content and revealed an about 8-fold higher and 5-fold deeper curcumin penetration than the nanocrystal formulation.

Despite the excellent performance of the smartFilms, the study revealed that the testing of particle containing dermal formulations can be misleading if conducted in infinite dose setup. The high volumes can cause particle sedimentation that will not occur in a real in vivo situation. The settled particles connect to the skin and/or the test membrane and lead to high local concentration gradients. This results in a high local penetration of the active, mimicking a high passive diffusion of the active ingredient through the skin or the test membrane.

Results of the study also provide evidence that particles suspended in a vehicle can directly interact with the skin. It is suggested that a liquid meniscus is formed in between the skin and the particles that come into close contact with the skin. The meniscus and the resulting capillary forces maintain the connection between particle and skin. Besides particle adhesion, the meniscus promotes passive dermal penetration by a local swelling of the stratum corneum and by the maintenance of a locally high concentration gradient of dissolved AI. Consequently, it is expected that menisci that cover large skin areas and that remain liquid on the skin for an extended time can promote dermal and transdermal penetration of active compounds most efficiently. The here described mechanism can be the base for the development of particle containing topical formulations for improved dermal and transdermal drug delivery. Further research is now needed to elucidate the newly proposed mechanism in more detail.

## Figures and Tables

**Table 1:** Overview of results obtained from the physicochemical characterization of curcumin-loaded smartFilms, bulk- and nanosuspensions.

	SmartFilms			Bulk Suspension Non-Diluted			Nanosuspension Non-Diluted			Bulk Suspension Diluted			Nanosuspension Diluted		
curcumin content [µg] in formulation	684	±	16	2098 *	±	41	1946 *	±	48	574 *	±	99	614 *	±	11
z-average [nm]	**n.a.**			**n.a.**			217	±	0.1	**n.a.**			205	±	0.1
PDI							0.31	±	0.02				0.35	±	0.02
d(v)0.1 [µm]				11	±	0.5	0.02	±	0.0	8	±	0.3	0.02	±	0.0
d(v)0.5 [µm]				45	±	7	0.1	±	0.0	24	±	2	0.1	±	0.0
d(v)0.9 [µm]				153	±	58	0.2	±	0.0	65	±	9	0.2	±	0.0
d(v)0.95 [µm]				188	±	77	0.5	±	0.0	84	±	15	0.5	±	0.0
d(v)0.99 [µm]				251	±	113	2.5	±	0.2	130	±	32	2.2	±	0.1
amount of curcumin [µg/cm^2^] applied on skin	152			466			432			128			136		

* Content of curcumin represents the amount of curcumin in 50 µL (corresponding volume of formulation applied on skin).

## Data Availability

The data presented in this study are available in Supplementary Materials.

## Supplementary Materials

The following are available online, S1: images of skin sections obtained from epifluorescence microscopy. S2: HPLC method validation report, Table S1: results from HPLC method validation, S3: automated macro-algorithms of digital imaging processing via ImageJ software.

## Sample Availability

Samples of the smartFilms, nanosuspensions, bulk suspensions and curcumin are available from the authors.
