# Supplementary material for: Improved Dermal and Transdermal Delivery of Curcumin with SmartFilms and Nanocrystals
Source: Molecules. 2021 Mar 15;26(6):1633. doi: 10.3390/molecules26061633 (PMC8000619; doi:10.3390/molecules26061633)
Supplement: Supplementary file 1 [file molecules-26-01633-s001.zip › molecules-1148497-supplementary.pdf]

molecules

MDPI

S1 – images of skin sections - untreated (A), treated with diluted bulk suspension (B), diluted nanosuspension (C), smartFilms (D), non-diluted bulk material (E) and non-diluted nanosuspension (F) – obtained by epifluorescence microscopy (magnification 200fold).

A: Blank – untreated skin

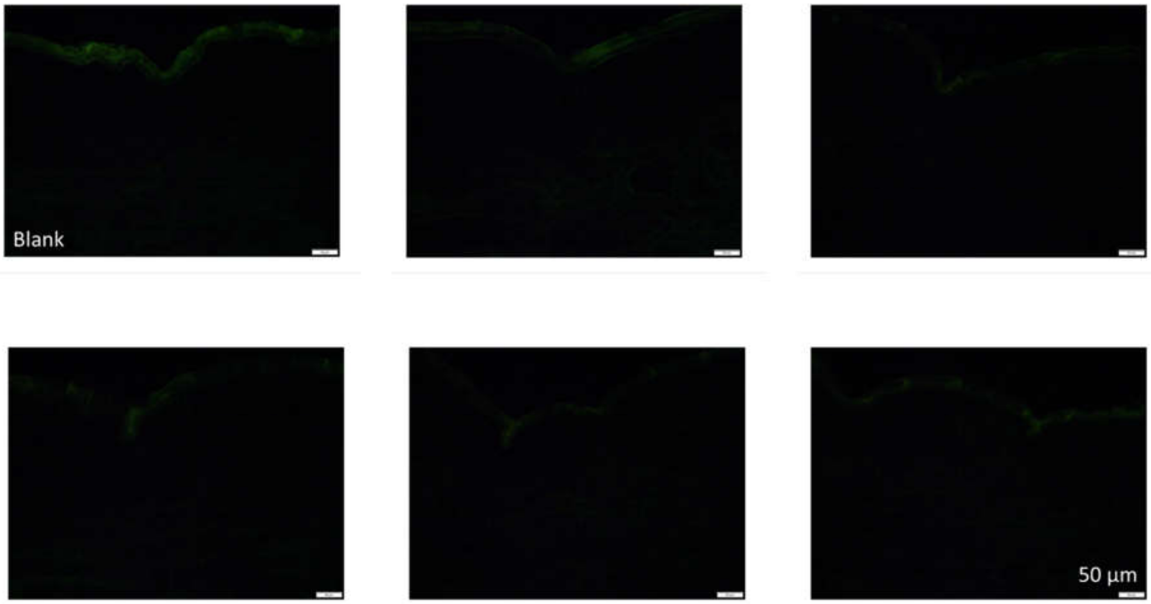

B: Bulk - diluted

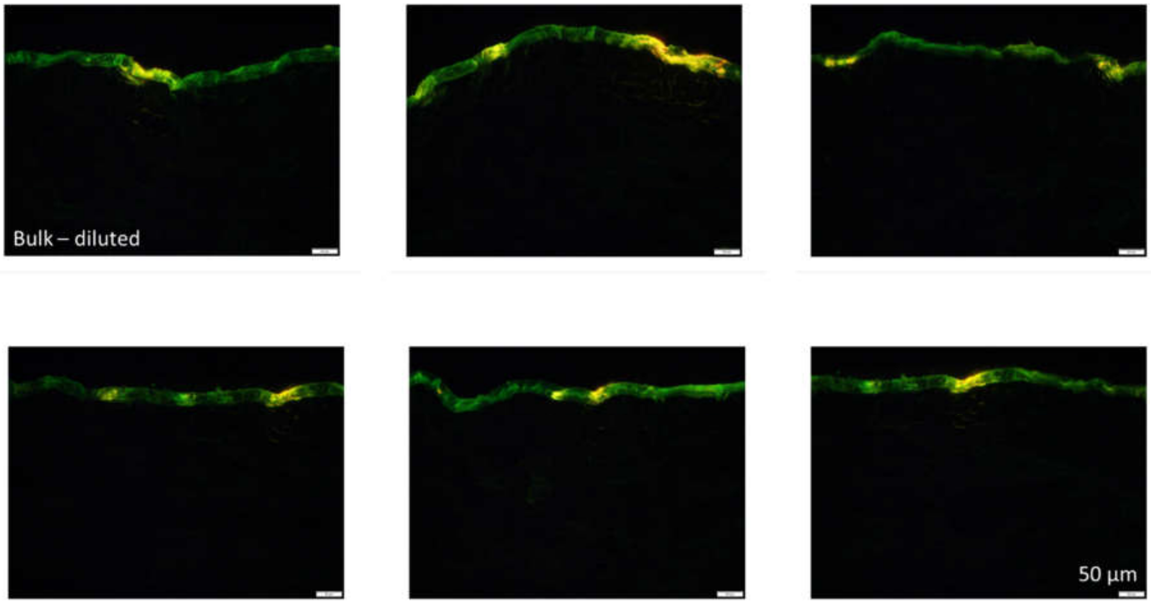

C: Nano - diluted

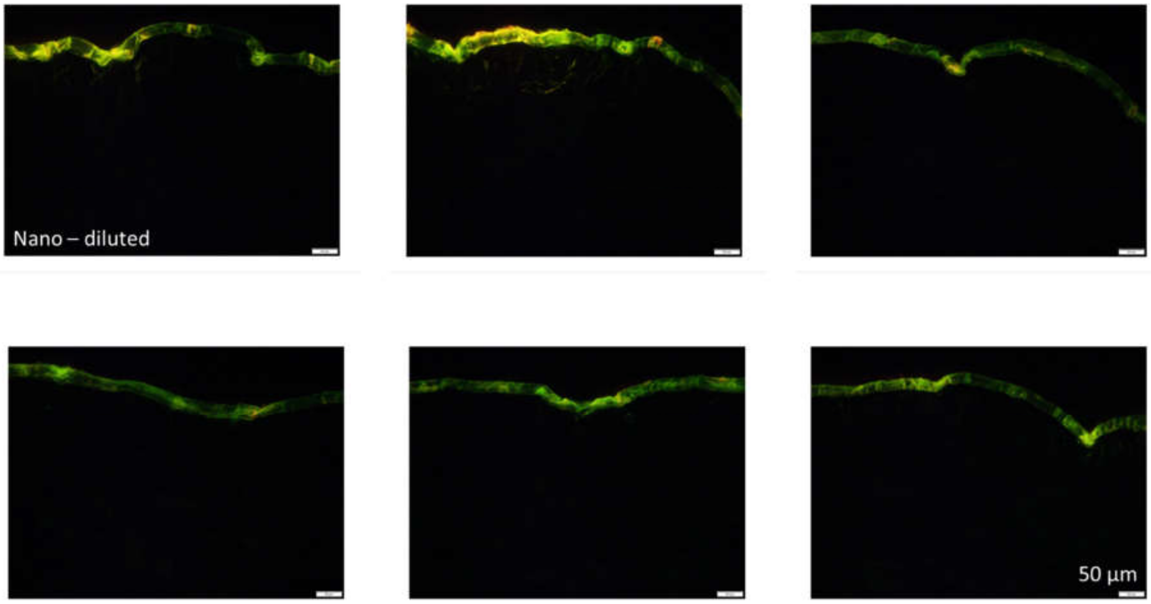

D: smartFilms

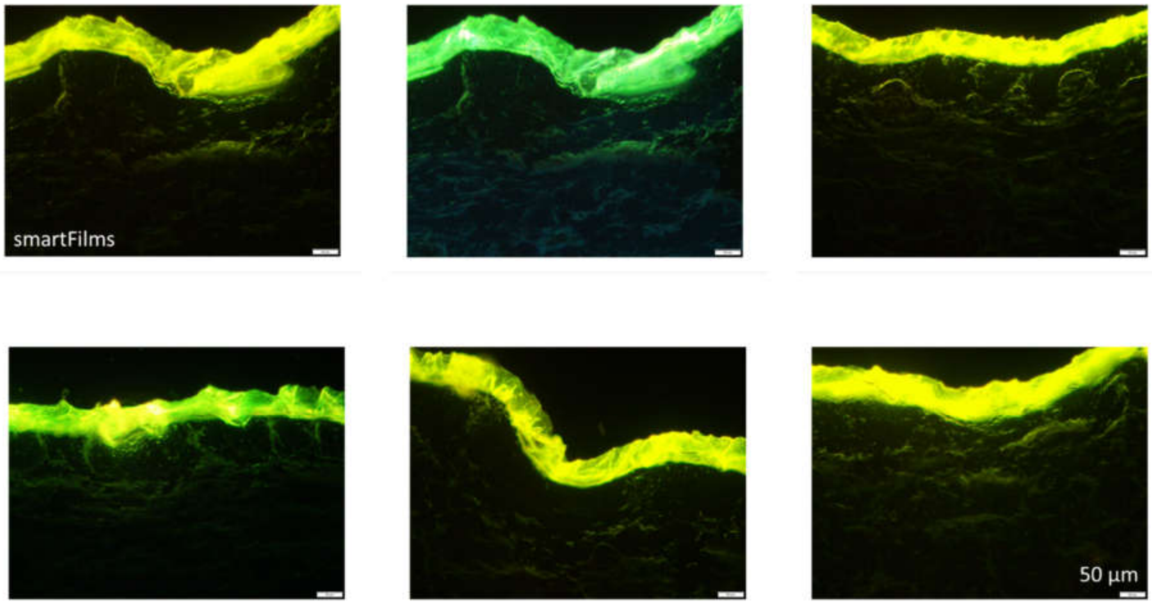

E: Bulk - non-diluted

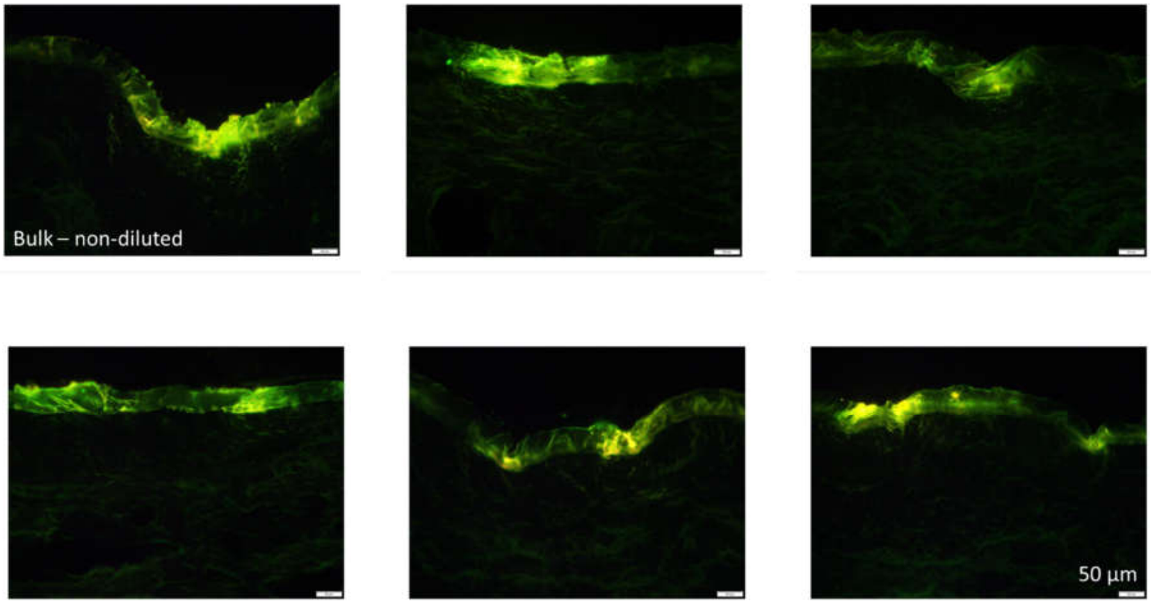

F: Nano - non-diluted

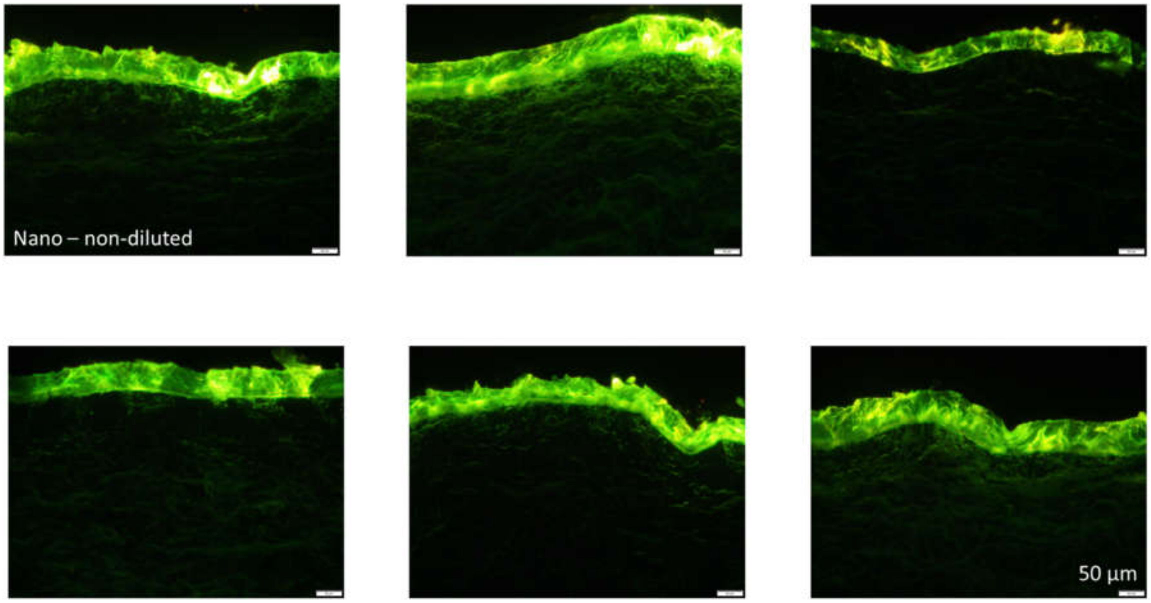

S2 – The Curcumin content was analysed with a validated HPLC method. The validation protocol is presented below.

Acceptance criteria for the HPLC method validation:

- range of the method was 0.125 µg/mL (= limit of quantification, LOQ) to 80 µg/mL
- seven-point calibration with a linear correlation coefficient of r2 = 0.9999
- Limit of detection (LOD) was 0.05 µg/mL
- LOD and LOQ were defined based on the signal-to-noise-ratio of 3 : 1 (LOD) and 10:1 (LOQ)
- intra-day and inter-day accuracy were evaluated using three different concentration levels (5 µg/mL, 10 µg/mL and 20 µg/mL) with an acceptance criterion in the range 98 – 102 % recovery
- precision was tested with six-fold injections of each calibration standard and the acceptance criterion was a relative standard deviation < 2 %
- robustness was determined with varying pH and ACN content of the mobile phase, flow rate and temperature. A curcumin standard solution with 10 µg/mL was used and the acceptance criterion was 98 – 102 % recovery

Results from the method validation

Table S1 shows the results of the HPLC method validation. It has to be noticed, that a lower ACN content of 35 % (v/v) exceeded the retention time of curcumin over the predefined run time of 10 minutes. Thus, the curcumin retention time is sensible to the organic content of the mobile phase and consequently the content was carefully monitored during preparation of the mobile phase. Furthermore, every HPLC sequence contained injections of the calibration standards at the beginning and the end and a lower ACN content would result in empty chromatograms. Additionally, a system suitability criterion for the HLPC sequence was defined. The peak area of the calibration standard at the beginning and the end of the sequence was compared and should only deviate within 2 % from another. Thus, the finding regarding the lower ACN content was considered as uncritical. The performance of the HPLC method for the quantification of curcumin was regarded as sufficient, since all other acceptance criteria were fulfilled.

Table S1. Results from HPLC method validation.

**intra-day**	**µg/mL**	**mean recovery [%]**	**RSD [%]**
	5	100.35	0.51
	10	99.12	0.30
	20	100.13	0.24
**inter-day**			
	5	99.43	0.53
**day 1**	10	98.29	0.31
	20	98.52	0.14
	5	98.57	0.37
**day 2**	10	99.51	0.26
	20	99.66	1.50
**robustness**			

**pH 1.90**	10 µg/mL	101.40	0.34
**pH 2.50**		98.94	0.40
**ACN 35 %**		run time exceeded	
**ACN 45 %**		101.40	0.15
**1.3 mL/min**		101.64	0.51
**1.5 mL/min**		99.89	0.92
**35 °C**		100.20	0.26
**45 °C**		99.15	0.16

S3 – digital imaging of the images was performed by ImageJ software and by subjecting all images to an auto-threshold mechanism to eliminate the autofluorescence of the skin from each image. The auto-threshold was set by two macros that were run subsequently for each image.

Macro 1 was:

```
// Color Thresholder 1.52a

// Autogenerated macro, single images only!

min=newArray(3);

max=newArray(3);

filter=newArray(3);

a=getTitle();

run("RGB Stack");

run("Convert Stack to Images");

selectWindow("Red");

rename("0");

selectWindow("Green");

rename("1");

selectWindow("Blue");

rename("2");

min[0]=0;

max[0]=0;

filter[0]="stop";

min[1]=33;

max[1]=255;

filter[1]="pass";

min[2]=0;

max[2]=0;

filter[2]="stop";

for (i=0;i<3;i++){

  selectWindow(""+i);

  setThreshold(min[i], max[i]);

  run("Convert to Mask");
```

```
  if (filter[i]=="stop")  run("Invert");
}
imageCalculator("AND create", "0","1");
imageCalculator("AND create", "Result of 0","2");
for (i=0;i<3;i++){
  selectWindow(""+i);
  close();
}
selectWindow("Result of 0");
close();
selectWindow("Result of Result of 0");
rename(a);
// Colour Thresholding-------------
```

Macro 2 was:

```
run("Invert");
```
